# [^18^F]FDG PET/CT in the Preoperative Diagnostic and Staging of Lung Cancer—A Pictorial Evaluation

**DOI:** 10.3390/jcm14134449

**Published:** 2025-06-23

**Authors:** Nathalie Viohl, Matthias Steinert, Anke Werner, Christian Kühnel, Martin Freesmeyer, Robert Drescher

**Affiliations:** 1 Clinic of Nuclear Medicine, Jena University Hospital, 07743 Jena, Germany; nathalie.viohl@med.uni-jena.de (N.V.); christian.kuehnel@med.uni-jena.de (C.K.); robert.drescher@med.uni-jena.de (R.D.); 2 Clinic of Thoracic Surgery, Leipzig University Hospital, 04103 Leipzig, Germany; matthias.steinert@medizin.uni-leipzig.de

**Keywords:** lung cancer, PET/CT, surgery, pitfalls, staging, TNM 9th edition

## Abstract

**Background**/**Objectives**: Lung cancer is one of the most prevalent malignant diseases in humans. Numerous studies have demonstrated the significance of [^18^F]fluorodeoxyglucose (FDG) positron emission tomography/computed tomography (PET/CT) in the staging of this condition. **Methods**: The pictorial evaluation is based on a recent study comparing preoperative imaging with postoperative histopathological findings following thoracic surgery. It confirmed the value of PET/CT in assessing primary tumor extent and metastatic lymph node involvement; but also revealed discrepancies in primary tumor (T) and lymph nodes (N) classification in 25% and 14% of patients, respectively. **Results**: The aim of this pictorial review is to highlight and further analyze the causes of inaccurate staging, identify potential diagnostic pitfalls, and provide practical recommendations to help avoid misinterpretation of PET/CT findings. Additionally, the impact of the newly introduced ninth edition of the International Association for the Study of Lung Cancer (IASLC) primary tumor, lymph nodes, and metastasis (TNM) staging system for lung cancer is discussed. **Conclusions**: In this pictorial review, we presented various sources of error in preoperative staging observed at our institution. Awareness of these potential pitfalls may aid in improving staging accuracy and distinguishing physiological or reactive (benign) processes from pathological findings.

## 1. Introduction

Positron emission tomography (PET) using [^18^F]fluorodeoxyglucose (FDG) to visualize glucose metabolism plays a critical role in the staging of primary lung carcinomas. Over time, it has been incorporated into international guidelines for the diagnosis and management of these tumors [1,2,3]. While PET/CT has been shown to provide high accuracy in staging lung cancer, postoperative histopathological findings do not fully correspond with imaging results in a relevant minority of cases [4,5,6,7,8,9]. It is therefore essential for both nuclear medicine physicians and thoracic surgeons to be aware of these limitations and to understand the underlying causes of such discrepancies.

The aim of this pictorial review is to highlight and further analyze the reasons for inaccurate staging, to identify potential diagnostic pitfalls, and to provide practical recommendations for avoiding misinterpretation of PET/CT findings. Additionally, the impact of the ninth edition of the International Association for the Study of Lung Cancer (IASLC) TNM staging system for lung cancer is discussed.

## 2. Materials and Methods

### 2.1. Patient Population

In the study “^18^F-FDG PET/CT in the preoperative diagnostic and staging of lung cancer and as a predictor of lymph node involvement” [10], a total of 104 patients who underwent whole-body FDG-PET/CT for lung cancer staging prior to thoracic surgery were analyzed. The median interval between PET/CT and surgery was 18.5 days (range: 1.0–113.0 days). PET imaging was acquired from the skull base to the upper thighs. An additional thoracic CT scan during deep inspiration was conducted to enhance the delineation of lung tissue.

PET/CT studies were evaluated by measuring the maximum standardized uptake values (SUV_max_) of pulmonary lesions, hilar and mediastinal lymph nodes, and any extrathoracic findings suspicious for metastasis. All patients underwent surgical staging, including thoracotomy, pulmonary resection, and thoracic lymphadenectomy. A pathological review was carried out to assess the characteristics of the primary tumor and lymph node involvement. Pathological (p)TNM staging was then assigned accordingly.

### 2.2. TNM Staging System

The ninth edition of the IASLC TNM staging system (TNM-9) has recently been published [11,12]. This updated system reflects emerging evidence that patients with metastases confined to a single N2 nodal station have a more favorable prognosis compared to those with involvement of multiple N2 nodal stations. Similarly, patients with extrathoracic metastases limited to a single organ system have a better prognosis than those with metastases in multiple extrathoracic organ systems [13,14,15].

Lymph node stations are defined according to the IASLC lymph node map [16]. The thoracic lymph node stations include nodes near the clavicle, along the trachea and bronchi, at the hilum of the lungs, beneath the carina, and in the anterior and posterior mediastinum. When staging lung cancer, an N0 situation is defined as no regional lymph node metastasis, an N1 situation is defined as metastasis in ipsilateral peribronchial and/or ipsilateral hilar and/or intrapulmonary lymph nodes, an N2 situation is defined as metastasis in ipsilateral mediastinal and/or subcarinal lymph node(s) (N2a: single N2 single station involvement / N2b: multiple N2 single station involvement), and an N3 situation is defined as metastasis in contralateral mediastinal, contralateral hilar, ipsilateral or contralateral scalene, or supraclavicular lymph node(s).

For the N2a/b classification, the number of involved lymph node stations is considered rather than the total number of affected lymph nodes.

Changes introduced in TNM-9 compared to TNM-8 are the following:

- N2 is subdivided into N2a/N2b, representing involvement of single/of multiple ipsilateral mediastinal or subcarinal lymph node station(s), respectively.
- M1c is subdivided into M1c1/M1c2, representing multiple extrathoracic metastases in a single/in multiple organ system(s), respectively.

These refinements result in potential (sub)stage reclassifications. Based on N2a or N2b status, T1N2 tumors (previously stage IIIA) may be downstaged to stage IIB, T2N2 tumors (previously stage IIIA) may be upstaged to stage IIIB, and T3N2 tumors (previously stage IIIB) may be downstaged to stage IIIA. T4N2 tumors remain classified as stage IIIB. Both M1c1 and M1c2 tumors continue to be categorized as stage IVB [11].

### 2.3. Correlation Between PET and Histopathological Findings

The image-based clinical (c)TNM stage was correlated with (p)TNM findings [10].

The accuracy of PET/CT for T classification was 75.0%. Overstaging occurred in 13.5% of cases, while 11.5% were understaged. For histopathologically confirmed pT1a/b/c, pT2b, and pT3 tumors, PET/CT was accurate in more than 84% of cases and in 68.8% of pT4 tumors. Among tumors classified as cT2a, 60% were correctly staged. Of the eight discordant cases in this subgroup, five were downstaged and three were upstaged following histopathological examination.

Nodal staging analysis included 99 patients; those who were suspected to have cN3 metastases but underwent only N1/N2 nodal sampling were excluded. The histopathological prevalence of lymph node metastases was 42.3%. PET/CT accurately assessed nodal status in 85.9% of cases, with equal rates of overstaging and understaging. PET/CT demonstrated the highest accuracy in excluding lymph node metastases, correctly identifying pN0 status in 90% of cases. Accuracy for pN1 and pN2 determination was 61.5% and 88.5%, respectively.

PET/CT correctly identified malignant lesions in the contralateral lung (M1a) in five patients. Extrapulmonary tumor lesions (M1b/c) did not undergo systematic histopathological evaluation.

In 78.2% of patients, the preoperative PET/CT-based disease stage and substage were confirmed by histopathological analysis. In 21.8%, staging discrepancies were identified postoperatively: ten patients were understaged and nine were overstaged by PET/CT. The majority of these discrepancies were observed in patients with stage II disease on PET/CT.

## 3. Results

### 3.1. Influence of the Ninth Edition of the IASLC TNM Staging System for Lung Cancer

**Figure 1:**
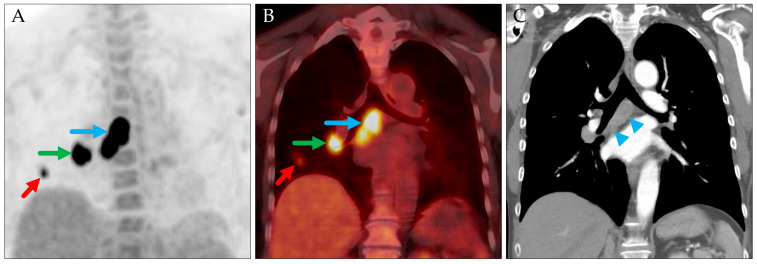
PET maximum intensity projection (MIP) in anterior–posterior (AP) view (**A**), coronal PET/CT (PET fused with attenuation-correction CT) (**B**), and coronal contrast-enhanced CT (**C**) acquired in the same session. The primary tumor is in the right lower lobe (red arrows; cT1b), right hilar (green arrows), and right subcarinal (blue arrows) lymph nodes (cN2). Contrast-enhanced CT confirms multiple nodes (blue arrowheads), but all are located within a single station (station 7) and are therefore are classified as cN2a according to TNM-9.

In the analyzed patient cohort, in only 1 of 99 patients did the stage designation change when using the TNM-9 system instead of the TNM-8 system. This patient’s tumor was classified as cT1b cN2 cM0, stage III, according to TNM-8. Under TNM-9, it was reclassified as cT1b cN2a cM0, stage IIB (Figure 1). In all other cases, the overall stage remained unchanged or only the substage was modified.

### 3.2. Incorrect Identification of Primary Tumor and Metastasis

**Figure 2:**
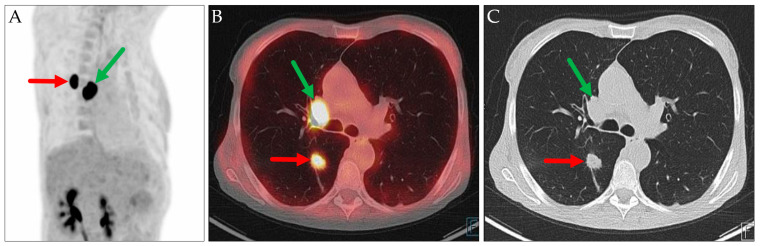
PET MIP: RAO view (**A**), axial PET/CT (**B**), and axial CT (**C**). Hypermetabolic lesions in the right lower lobe (slightly spiculated, red arrows) and at the right hilum (green arrows) described as a peripheral lung carcinoma (cT1c) with a single ipsilateral hilar metastasis (cN1). Histopathological evaluation showed a hilar adenocarcinoma with an intrapulmonary metastasis in the same lobe (pT3) and a metastatic lymph node directly adjoining to the primary tumor (pN1).

This following case highlights two limitations of PET/CT imaging (Figure 2). First, when the primary tumor is confluent with metastatic lymph nodes, PET/CT may be unable to differentiate between them, potentially leading to an inaccurate preoperative T staging. Second, PET/CT does not reliably distinguish between a primary tumor and a pulmonary metastasis. Consequently, it may be appropriate to report alternative cTNM staging scenarios for such patients.

### 3.3. Assessment of a Lung Carcinoma as a Pulmonary Metastasis of Another Primary Tumor

**Figure 3:**
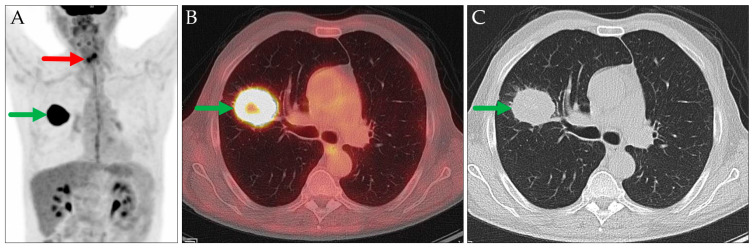
PET MIP: AP view (**A**), axial PET/CT (**B**), and axial CT (**C**). Cervical hypermetabolic area, corresponding to the patient’s known hypopharyngeal carcinoma (red arrow). Assumed pulmonary metastasis in the right lung (green arrows), which was histologically proven to be a primary lung carcinoma (pT3N0M0, stage IIA in TNM-8/9).

**Figure 4:**
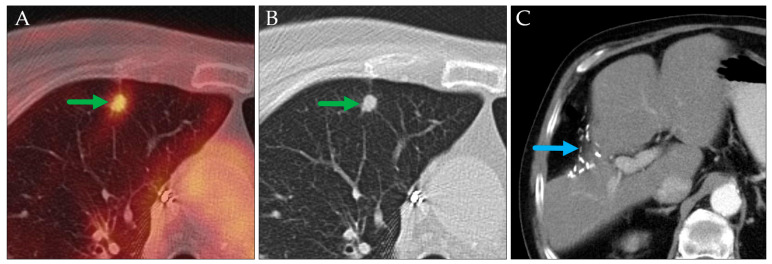
Axial PET/CT (**A**) and CT of the chest and abdomen (**B**,**C**). The condition after resection of a hepatocellular carcinoma (clips, blue arrow). Assumed solitary pulmonary metastasis is seen on the PET/CT (green arrow), which was histologically proven to be a primary lung carcinoma (pT1a pN0 c M0, stage IA1 in TNM-8/9).

In two cases involving patients with known extrapulmonary hypermetabolic malignancies, pulmonary lesions were initially presumed to represent metastases from the primary tumors based on their typical radiologic appearances (Figure 3 and Figure 4). Histopathological examination revealed that these lesions were instead primary lung carcinomas, representing an additional malignancy.

### 3.4. Lymph Node Involvement Underestimated Due to Low Glucose Metabolism of the Lymph Nodes

**Figure 5:**
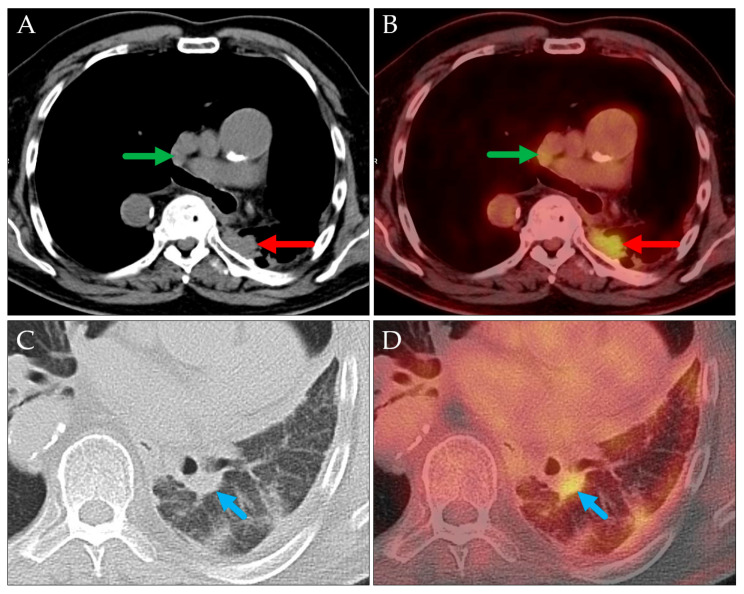
Axial CT (**A**,**C**) and PET/CT (**B**,**D**). Mediastinal shift to the left due to a hypoplastic left lung and left pulmonary artery. The primary tumor (SUV_max_ 4.8) with pleural infiltration (red arrows; cT3). A slightly hypermetabolic mediastinal lymph node (green arrows; short axis diameter 9 mm, SUV_max_: 2.9) was not considered metastatic on PET/CT (cN0) but was histologically proven to be malignant (pN2). A suspected ipsilateral metastasis in the same lobe (segment 10, blue arrows) was not malignant.

In the following case, PET/CT underestimated the extent of lymph node involvement, as the affected nodes were not pathologically enlarged and exhibited only marginally increased glucose metabolism (Figure 5). Consequently, they were interpreted as benign (cN0). However, histopathological analysis confirmed metastatic involvement (pN2). The T classification was consistent (cT3, pT3), though based on different criteria: the clinical T3 classification (cT3) was assigned due to a suspected metastasis in the same lobe, located caudally to the primary tumor, whereas the pathological classification (pT3) was based on evidence of parietal pleural infiltration. A study by Endoh et al. also demonstrated that low SUV_max_ values (<4) in involved lymph nodes are a significant factor contributing to the underestimation of nodal staging [17].

**Figure 6:**
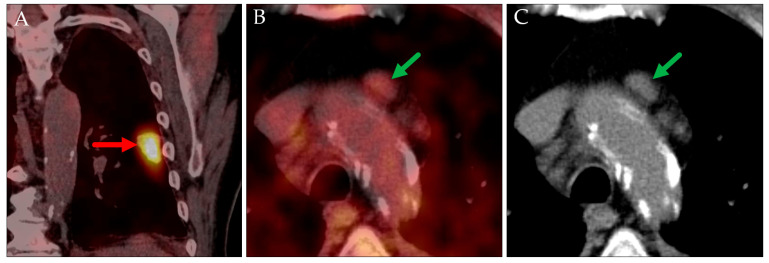
Coronal PET/CT (**A**), axial PET/CT (**B**), and axial CT (**C**). The primary tumor showed an intense glucose metabolism (SUV_max_ 20.1, red arrow). A slightly enlarged mediastinal lymph node (11 mm, green arrow) that was considered to be benign due to its low metabolism (SUV_max_ 2.1) and the missing evidence of malignant hilar lymph nodes (cN0). It was histologically proven as malignant (pN2).

In another case, PET/CT also underestimated the lymph node staging (Figure 6). Although the affected lymph nodes were morphologically slightly enlarged and the primary tumor exhibited markedly increased glucose metabolism, the lymph node themselves did not demonstrate elevated metabolism and was thus interpreted as benign. Additionally, the T classification was inconsistent, as the tumor size was underestimated on PET/CT (max. diameter 34 mm: cT2a; max diameter 45 mm: pT2b).

### 3.5. Mismatch Between Imaging and Histological Tumor Size

In several cases, the tumor size assessed by PET/CT did not correspond with histopathological measurements, leading to incorrect tumor classification in twelve patients. This included seven cases of overestimation and five of underestimation. One contributing factor to tumor size overestimation on PET/CT was the confluent appearance of the primary tumor with adjacent (malignant) lymph nodes (Figure 2). Due to their increased metabolic activity, these lymph nodes could not always be reliably distinguished from the primary tumor and thus this artificially inflated the measured tumor size.

**Figure 7:**
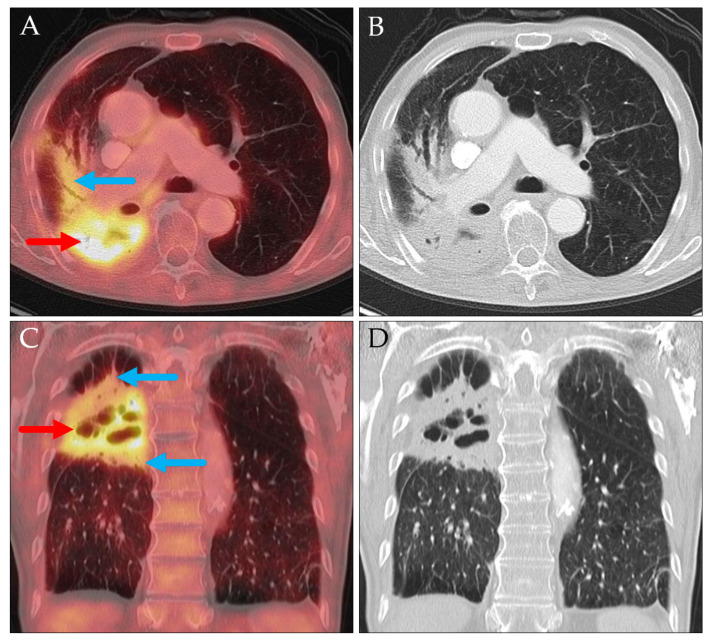
Axial PET/CT (**A**) and CT (**B**); coronal PET/CT (**C**) and CT (**D**). Primary lung carcinoma (red arrows) with peritumoral infiltrates/atelectases (blue arrows), which are also hypermetabolic and difficult to differentiate from actual tumor tissue.

**Figure 8:**
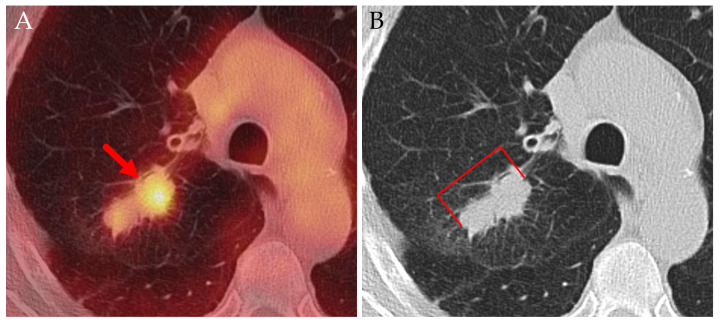
On axial PET/CT (**A**) and CT (**B**), the size of the primary tumor was measured as 3.5 cm (red arrow and bracket, cT2a). The histological tumor size was 2.1 cm (pT1c) with an adjacent atelectasis.

In other cases, accurate measurement of tumor diameter was hindered by ill-defined, peritumoral inflammatory or infiltrative changes, which also exhibited increased glucose metabolism. Such circumstances were observed, for example, in tumors with diffuse extensions or in the presence of surrounding atelectasis (Figure 7 and Figure 8).

Reasons for the underestimation of tumor size on PET/CT were that the regions initially suspected to represent atelectasis due to their low glucose metabolism were actually part of the primary tumor, and also that tumor growth progressed in the interval between PET/CT imaging and surgical intervention (see 3.8).

### 3.6. Incorrect T Classification Due to Granulomas

**Figure 9:**
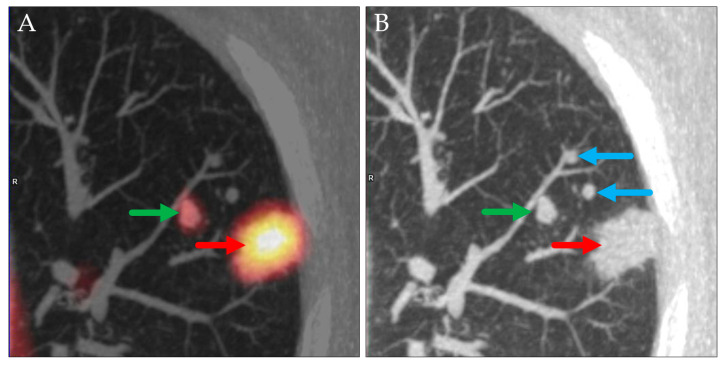
Axial PET/CT (**A**) and CT (**B**). The primary tumor infiltrating the visceral pleura (SUV_max_ 14.0, red arrows). A nodule next to it was evaluated as a satellite metastasis (SUV_max_ 1.7, green arrows; cT3). All small nodules were later diagnosed to be granulomas (green/blue arrows; pT2a).

A factor contributing to overestimation of the T classification was the misinterpretation of presumed satellite metastases, which were later histologically identified as granulomas. These lesions exhibited increased glucose metabolism on PET/CT due to underlying inflammatory reactions, leading to their incorrect classification as malignant (Figure 9).

### 3.7. Mismatch in Nodal Staging Due to Inflammatory Consolidations

**Figure 10:**
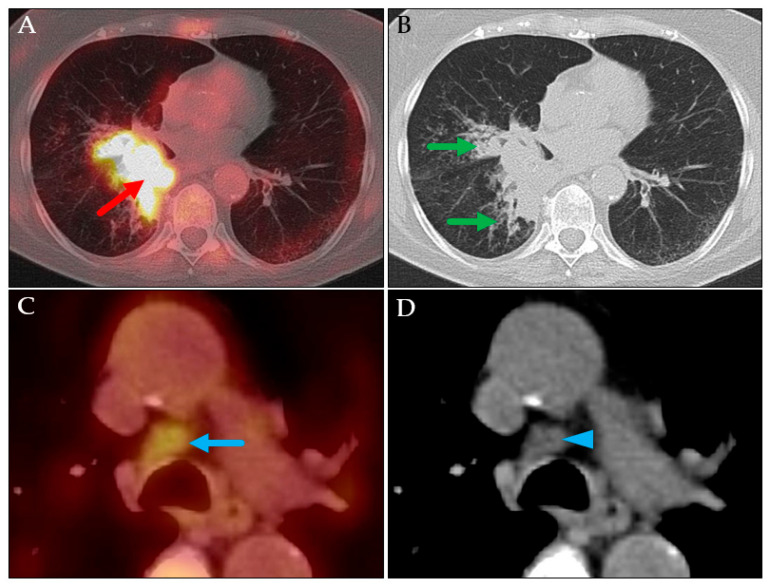
Axial PET/CT (**A**,**C**) and CT (**B**,**D**). A highly hypermetabolic primary tumor (red arrow, cT3 due to infiltration of the visceral pleura) with peritumoral infiltrates (green arrows). An enlarged mediastinal lymph node (12 mm) was reported to be malignant (blue arrow; SUV_max_ 5.8, cN2, cN2a according to TNM-9). The node had a small fatty hilum as a sign of benignity (blue arrowhead), but the metabolism appeared suspicious. Histopathologically it was proven to be inflammatory (pN0).

**Figure 11:**
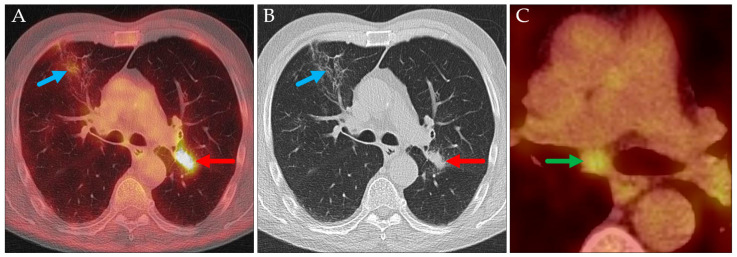
Axial PET/CT (**A**,**C**) and CT (**B**). The primary tumor (cN1, red arrow; cT2a) and florid contralateral infiltrates (blue arrows, SUV_max_ 2.8). The suspected mediastinal lymph node metastasis (green arrow; SUV_max_ 4.3, short axis diameter 11 mm; cN2; cN2a according to TNM-9) was histopathologically proven to be pN0.

Preoperative nodal staging in patients with pneumonic or peritumoral infiltrates—potentially resulting from bronchial compression by a centrally located tumor (Figure 10) or even when located in the contralateral lung (Figure 11)—can be challenging. In such scenarios, lymph node involvement was overestimated in four cases, accounting for 28.6% of all overestimations in this study. In these cases, a general recommendation can be made: both pathologically enlarged lymph nodes (>1 cm in short-axis diameter) and nodes exhibiting slight to moderate hypermetabolism should be interpreted with caution. Rather than being classified as malignant, such findings may more appropriately reflect reactive changes. A study by Endoh et al. similarly reported that lymph node involvement was more frequently overestimated in the context of inflammation [17].

### 3.8. Progression Between Preoperative PET/CT and Surgery

Tumor progression during the interval between preoperative PET/CT and surgical resection was also identified as a potential factor negatively impacting the accuracy of preoperative staging. In our patient cohort, no statistically significant influence could be identified because in less than 10% of patients, the interval was longer than 8 weeks (median 18.5 days, range 1.0–113.0 days). In these 10 cases, two T staging and one N staging discrepancies were detected, respectively.

**Figure 12:**
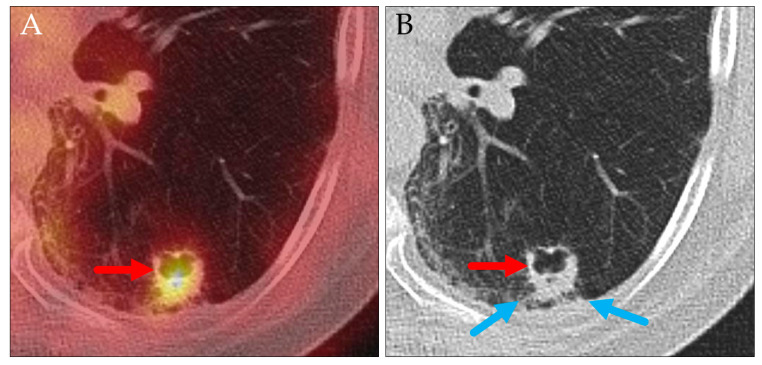
Axial PET/CT (**A**) and CT (**B**) of a patient with squamous cell carcinoma. Despite the small spiculae reaching the visceral pleura (blue arrows), the partially necrotic primary tumor (red arrows) was classified as cT1b. At the time of surgery more than three months later, a broad invasion of the visceral pleura was found (pT2a).

In one case of squamous cell carcinoma, infiltration of the visceral pleura over a 109-day period resulted in an upstaging from cT1b to pT2a (Figure 12). In another case, also involving squamous cell carcinoma, tumor growth over 90 days led to an increase in size from 63 mm (cT3) on PET/CT to 80 mm (pT4) at the time of surgery.

Conversely, in a patient diagnosed with adenocarcinoma, no change in TNM classification was observed despite an interval of 113 days between PET/CT and resection.

**Figure 13:**
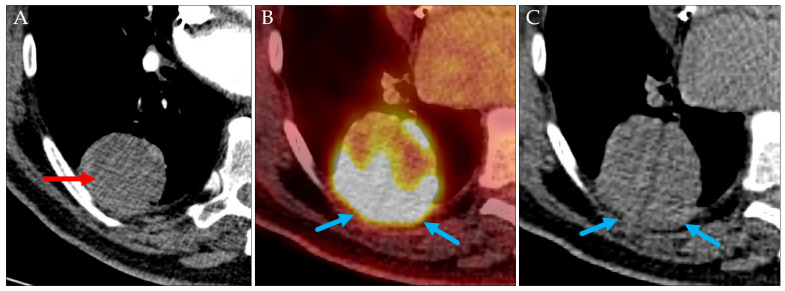
The initial CT (**A**; axial, contrast-enhanced) revealed a 5.2 cm mass in the right lower lobe abutting the pleura (red arrow; cT3). Axial PET/CT (**B**) and CT (**C**) performed 28 days later showed size progression to 6.4 cm and infiltration of the parietal pleura (blue arrows, still cT3).

In another case involving a combined small cell lung carcinoma (SCLC) and non-small cell lung carcinoma (NSCLC) of grade 3, tumor progression was observed despite a short interval of only 13 days between PET/CT and surgery. This progression led to an upstaging from cT3 to pT4 (Figure 13). This case supports the observation that different tumor types and histological grades exhibit varying rates of progression [18]. Based on clinical experience at our center, it is recommended that preoperative staging with PET/CT be performed as close to the time of surgery as possible—ideally within a maximum interval of three months, and preferably within a much shorter timeframe.

## 4. Discussion

Based on the cases evaluated in this study, several factors contributing to discrepancies between preoperative and postoperative TNM classification were identified:

- Misclassification of a primary lung carcinoma as a pulmonary metastasis from another primary tumor.
- Inaccurate measurement when the primary tumor is confluent with (malignant) lymph nodes.
- Measurement discrepancies between PET/CT and histopathology due to differing methodologies and subjectivity.
- Prolonged interval between preoperative PET/CT and surgery, allowing for tumor progression.
- False-positive findings caused by benign conditions such as inflammatory reactions or granulomas.
- False-negative findings due to low SUV_max_ in lymph nodes or the primary tumor.

Recommendations derived from these findings include the following:

- Preoperative PET/CT should be performed in all cases of lung carcinoma.
- The interval between PET/CT and surgery should be minimized to ensure imaging reflects the current extent of disease, particularly in tumors with rapid growth.
- In cases of diagnostic uncertainty, alternative cTNM staging scenarios should be considered and reported, taking into account the overall clinical context. Since TNM staging systems may be updated every few years, the most recent version (for lung cancer: IASLC TNM version 9) [12] has to be used and differences reported when necessary.
- In the presence of pneumonic or peritumoral infiltrates, or in cases of ventilation impairment (e.g., due to bronchial compression by a centrally located tumor), both pathologically enlarged lymph nodes (>1 cm in short-axis diameter) and lymph nodes with mildly to moderately increased glucose metabolism should be interpreted cautiously. These may more appropriately represent reactive changes rather than malignant involvement.

Despite these potential sources of error, discrepancies between PET/CT and histopathological measurements remain unavoidable to some extent, as the two methods are based on fundamentally different principles. Moreover, tissue processing procedures—such as surgical resection, fixation, and specimen handling—occur between imaging and final pathological assessment, potentially contributing to variations in the measured tumor size.

## 5. Conclusions

FDG-PET/CT has become an invaluable tool in the staging of lung cancer. In this pictorial review, we presented various sources of error in preoperative staging observed at our institution. Awareness of these potential pitfalls may aid in improving staging accuracy and distinguishing physiological or reactive (benign) processes from pathological findings.

## Data Availability

The original contributions presented in this study are included in the article. Further inquiries can be directed to the corresponding author(s).
